# Exploring the histopathological signature of repeat‐mediated Fuchs endothelial corneal dystrophy

**DOI:** 10.1111/aos.70014

**Published:** 2025-10-14

**Authors:** Anne‐Marie S. Kladny, Nihar Bhattacharyya, Marcos Abreu Costa, Stephen J. Tuft, Caroline Thaung, Alice E. Davidson

**Affiliations:** ^1^ UCL Institute of Ophthalmology London UK; ^2^ Eye Centre Albert‐Ludwigs‐University of Freiburg Freiburg Germany; ^3^ Moorfields Eye Hospital London UK

**Keywords:** corneal dystrophy, CTG18.1, Fuchs endothelial corneal dystrophy, genotype–phenotype, guttae, histopathology, repeat expansion, *TCF4*, triplet‐repeat

## Abstract

**Purpose:**

To determine the histological differences between Fuchs endothelial corneal dystrophy (FECD) cases with and without the most common genetic risk factor, expansion of a CTG repeat (CTG18.1) within the *TCF4* gene.

**Methods:**

Formalin‐fixed paraffin‐embedded corneal tissues were compared retrospectively, and CTG18.1 status was determined from blood‐derived gDNA. Cases were defined as expansion‐positive (Exp^+^) if at least one CTG18.1 allele had ≥ 50 repeats. Tissue was assigned as either ‘typical’ if there were several prominent exophytic or buried guttae or ‘not typical’ if there were only a few shallow guttae or no status could be confidently assigned.

**Results:**

In total, 72 unrelated corneal specimens (43 from endothelial keratoplasty and 29 from penetrating keratoplasty) with corresponding genetic data were analysed. We assigned a ‘typical’ histopathological guttae appearance to 88% (53/60) of Exp^+^ and 83% (10/12) of Exp^−^ cases. No significant difference was observed between the proportion of ‘typical’ and ‘not typical’ assignments within these genotypically distinct groups (Fisher's exact test; odds ratio [OR], 1.5; 95% CI: 0.14–9.7; *p* = 0.6393). Without adjustment for multiple testing, ‘not typical’ samples were more likely to be from males (7/9 vs. 17/63; OR, 0.11; 95% CI: 0.01–0.65; *p* = 0.005017) and more frequently from cases with coexisting keratoconus (4/9 vs. 3/63; OR = 0.07; 95% CI: 0.01–0.54; *p* = 0.004208).

**Conclusion:**

Histopathological guttae appearance was not found to correlate with CTG18.1 expansion status. However, male sex or coexisting keratoconus was more frequently associated with histology categorised as ‘not typical’.

## 1 INTRODUCTION

Fuchs endothelial corneal dystrophy (FECD) is the leading cause of corneal transplantation in high‐income countries (Mathews et al., 2023). In affected individuals, the corneal endothelial cells (CECs) show morphological changes and degeneration with accelerated apoptosis. Irregular thickening of the Descemet membrane (DM) and subendothelial deposits (guttae) are hallmarks of the disease (Matthaei et al., 2019; Vogt, 1930). The guttae predominantly affect the central cornea and are composed of short fibrillar type VIII collagen and other extracellular matrix (ECM) proteins (Matthaei et al., 2019). The histopathological appearances of the DM can be variable (Chi et al., 1958; Yuen et al., 2005). Generally, the guttae are either exophytic or buried within a posterior fibrillar layer (Thaung & Davidson, 2022). In addition, some cases have no histopathological evidence of guttae despite apparent guttae at clinical examination (for example, (Hogan et al., 1974) and (Gottsch, Sundin, et al., 2005; Gottsch, Zhang, et al., 2005)).

Understanding of the genetic causes of FECD has dramatically advanced over the past decades (Fechner et al., 2025). The majority of FECD cases with predominantly European ancestry harbour an expansion of a non‐coding triplet repeat (≥ 50 CTG repeats within CTG18.1) within the *TCF4* gene (Exp^+^) (Liu et al., 2025; Viberg et al., 2022; Wieben et al., 2012). A much smaller proportion of disease is attributed to gain‐of‐function missense variants in *COL8A2*, the gene encoding for the alpha 2 chain of type VIII collagen (Biswas et al., 2001), which can give rise to a clinically distinct early‐onset form of FECD (Gottsch, Sundin, et al., 2005; Gottsch, Zhang, et al., 2005; Liskova et al., 2007). Our recent multicentre cohort study confirmed that causative *COL8A2* pathogenic variants affect only a minority (< 0.3%) of FECD cases and that most cases without a CTG18.1 expansion (Exp^−^) are genetically unresolved (Liu et al., 2025). Histopathologically, FECD cases harbouring *COL8A2* pathogenic variants have a distinct appearance with small or absent guttae, even in advanced disease (Magovern et al., 1979; Gottsch, Sundin, et al., 2005; Gottsch, Zhang, et al., 2005; Zhang et al., 2006). To date, *COL8A2* pathogenic variants are the only genetic subtype of FECD reported to have a distinctive histopathological phenotype.

Despite advances in our understanding of FECD genetic architecture, the histological signature of CTG18.1 expansions (Exp^+^) disease, the most common genetic subtype, has, to the best of our knowledge, not been investigated. In this study, we compare the histological guttae appearance of CTG18.1 Exp^+^ and Exp^−^ FECD.

## 2 MATERIALS AND METHODS

### 2.1 Participants and baseline characteristics

All participants had a clinical diagnosis of FECD documented before a primary penetrating keratoplasty (PKP) or endothelial keratoplasty (EK) performed at Moorfields Eye Hospital (MEH) (Figure S1 for exclusion/inclusion criteria). The excised corneal tissue was processed by the Department of Eye Pathology (MEH). Blood‐derived gDNA samples were used for genetic testing. Patient records were reviewed retrospectively, and data were extracted on any eye or systemic diseases that could affect the cornea. Individuals with other diseases affecting the cornea, like keratoconus (KC) or glaucoma, were included unless unusual additional corneal changes occurred that could not be clearly defined. The diagnosis of KC was based on documented clinical diagnosis according to established criteria. Part of our cases was described earlier by Liu et al. (2023), where we delineated the diagnostic criteria to confirm keratoconus in the presence of FECD. Age at first keratoplasty was used as a surrogate for disease severity. If corneal specimens from both eyes were available, we only included the specimen from the first surgery for genotype–phenotype analysis (Figure S1). However, both specimens were individually investigated histopathologically, and their appearance was compared. The study adhered to the tenets of the Declaration of Helsinki. It was approved by the Research Ethics Committees of University College London (UCL) (22/EE/0090) or Moorfields Eye Hospital (MEH) London (13/LO/1084). All participants provided written informed consent.

### 2.2 DNA extraction and CTG18.1 genotyping

DNA was extracted from peripheral blood leucocytes using a Gentra® Puregene® kit (Qiagen). DNA samples were analysed using a short tandem repeat‐polymerase chain reaction assay to detect and size CTG18.1 alleles (Zarouchlioti et al., 2024). FECD cases were defined as expansion‐positive (Exp^+^) if at least one CTG18.1 allele had ≥ 50 CTG repeats. For all expansion‐negative (Exp^−^) cases, *COL8A2* was screened for the known causative pathogenic variants c.1349T>G, p.(Leu450Trp) and c.1363C>A, p.(Gln455Lys) (Biswas et al., 2001; Gottsch, Sundin, et al., 2005; Gottsch, Zhang, et al., 2005) using conventional PCR and Sanger sequencing with forward primer 5′‐GTGACCAGGGGCCTAGTG‐3′ and reverse primer 5′‐CCTGCGATGCCAGTCTCAT‐3′ (Biswas et al., 2001). In a subset of the Exp^−^ cases, exome sequencing and variant annotation were additionally performed as part of our previous cohort study (Liu et al., 2025) using SureSelect Human All Exome V6 capture kit (Agilent, UK) and sequenced on HiSeq 4000 platform (Illumina, UK). Raw sequencing data were aligned to the Genome Reference Consortium Human Build 38 patch release 33 (GRCh38.p13) using Burrows‐Wheeler Aligner v0.7.17 16 (Li & Durbin, 2009; Pontikos et al., 2017). Variants and indels were called according to the Genome Analysis Toolkit Haplotypecaller v4.417 (DePristo et al., 2011) and annotated using Ensembl Variant Effect Predictor v106.1 18 (McLaren et al., 2016) alongside Combined Annotation Dependent Depletion (CADD) v1.619 (Rentzsch et al., 2019). Where possible, ancestry was determined using principal component analysis of genome‐wide single‐nucleotide polymorphism (SNP) array data (FRAPOSA) (Zhang et al., 2020) as previously reported (Liu et al., 2025).

### 2.3 Histological analysis

Corneal samples were formalin‐fixed and paraffin‐embedded following standard protocol. Four µm sections were stained with haematoxylin and eosin (H&E) and periodic acid‐Schiff (PAS) and analysed by light microscopy. To evaluate the reproducibility of subjective guttae grading, each sample was assessed by an experienced ocular histopathologist (rater 1, CT) and an ophthalmologist (rater 2, ASK) newly trained to identify the histological changes of FECD. Both observers were masked to the genotype. Rater 1's scores were used throughout for analysis.

**FIGURE 1:**
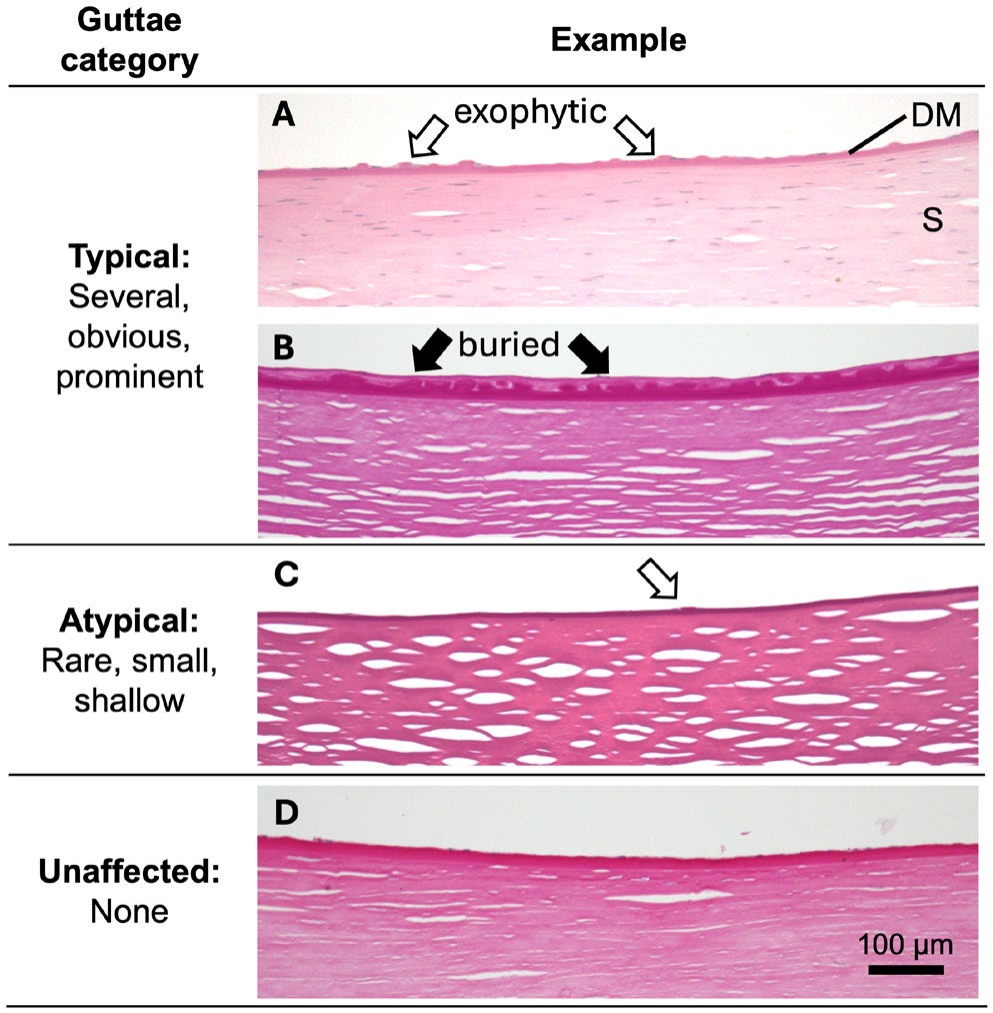
Definition and examples of different histopathological guttae categories in Fuchs endothelial corneal dystrophy corneal tissues utilised in this study. Periodic acid‐Schiff‐stained sections of corneal stroma (S), Descemet membrane (DM) and endothelium, 20× objective magnification. Allocation to ‘typical’ (a, b) or ‘atypical’ (c) guttae category depended on the amount and size of the guttae at the DM. ‘Intermediate’ category included cases that could not confidently be assigned to either the ‘typical’ or the ‘atypical’ category. Exophytic guttae (white arrows) are protrusions on the posterior surface of the cornea. Buried guttae (black arrows) are covered by a membrane structure, leading to a reasonably smooth posterior corneal surface. An unaffected cornea (d) shows no sign of guttae. Scale bar = 100 μm.

Samples were assigned as ‘typical’, ‘atypical’, or ‘intermediate’ based on the appearance of the guttae. A ‘typical’ guttae appearance was defined as several prominent exophytic or buried guttae; an ‘atypical’ guttae appearance was defined as rare and shallow guttae (Thaung & Davidson, 2022). Specimens that could not confidently be assigned to either group were defined as ‘intermediate’ (Figure 1). For statistical analysis, we amalgamated the ‘atypical’ and ‘intermediate’ categories as ‘not typical’. The main outcome measure was the correspondence between *TCF4* expansion status (Exp^+^ or Exp^−^) and histopathological grading.

### 2.4 Statistical analysis for genotype–phenotype comparisons

After assessing the data distribution for normality, we analysed the correspondence between the other patient characteristics and histopathological categories using Fisher's exact test for categorical variables or Mann–Whitney *U* test for continuous variables. A difference with *p* < 0.05 was considered statistically significant. Metric values are presented as mean with standard deviation for normally distributed variables, and for other variables as median and range, and categorical variables as frequency and percentage. Inter‐rater reliability was assessed using Cohen's kappa. Statistical analysis was performed using R (version 2023.12.1 + 402).

## 3 RESULTS

### 3.1 Baseline characteristics and epidemiological features

In total, 72 FECD patients from our larger FECD cohort were included in this study. Overall, they were deemed to be representative of the total FECD patient cohort recruited at MEH (Liu et al., 2025) across several categories, including CTG18.1 expansion status, ancestry, and sex. Sixty patients (83%) harboured one or more expanded CTG18.1 alleles, and the remaining 12 harboured non‐expanded CTG18.1 alleles.

Within this genetically unresolved Exp^−^ group, all cases were also confirmed to be negative for causative *COL8A2* variants (Biswas et al., 2001; Meng et al., 2013). In 10 out of the 12 Exp^−^ cases, additional exome sequencing was performed and did not show any other potentially pathogenic variants (MAF < 0.005, CADD > 10) in *COL8A2* or other FECD associated genes (*SLC4A11*, *ZEB1*, and *TCF4*). Overall, 82% (59/72) of individuals included in this study were of European ancestry. The female to male ratio was 2:1. However, the female skew was notably higher in the Exp^−^ group (5:1) compared with the Exp^+^ group (1.7:1). Median age at first keratoplasty was similar between the Exp^+^ and Exp^−^ participants (67 years (range, 33–87) vs. 67 years (range, 32–81)), although Exp^−^ participants in our larger cohort study were slightly younger (Liu et al., 2025). In this study, 21/60 (35%) of the Exp^+^ corneal specimens and 8/12 (67%) of the Exp^−^ specimens were from a PKP, while the remaining tissue specimens were from EK. In both groups, cataract surgery had been performed in 33% of the cases before keratoplasty. The most common corneal comorbidity was KC, affecting 5/60 (8%) individuals in the Exp^+^ group and 2/12 (17%) in the Exp^−^ group (Table 1). Of those, one Exp^+^ and one Exp^−^ case were already reported as part of an earlier study (Liu et al., 2023).

**TABLE 1:** Participant characteristics by genotype: expansion‐positive (Exp^+^) versus expansion‐negative (Exp^−^) Fuchs endothelial corneal dystrophy.

	Exp^+^	Exp^−^
Participants, *n* (eyes)	60 (60)	12 (12)
Female, *n* (%)	38 (63)	10 (83)
Ancestry^a^		
European, *n* (%)	51 (85)	8 (67)
African, *n* (%)	2 (3)	1 (8)
South Asian, *n* (%)	0 (0)	1 (8)
Not known, *n* (%)	7 (12)	2 (17)
Age at first keratoplasty, years, median (range)	67 (33–87)	67 (32–81)
Age at tissue sample collection, years, median (range)	68 (33–87)	66 (32–81)
Type of surgery		
Penetrating keratoplasty, *n* (%)	21 (35)	8 (67)
Endothelial keratoplasty, *n* (%)	39 (65)	4 (33)
Keratoplasty only		
Phakic throughout surgery, *n* (%)	10 (17)	2 (17)
Pseudophakic throughout surgery, *n* (%)	20 (33)	4 (33)
Combined cataract surgery, *n* (%)	28 (47)	6 (50)
Lens status not documented, *n* (%)	2 (3)	0 (0)
Year of surgery, median (range)	2016 (1998–2023)	2012 (2003–2023)
Additional eye conditions with effect on the cornea before surgery		
None, *n* (%)	49 (78)	8 (67)
≥1 additional eye condition, *n* (%)	11 (18)	4 (33)
Keratoconus, *n* (%)	5 (8)	2 (17)
Glaucoma, *n* (%)	4 (7)	1 (8)
Keratitis, *n* (%)	3 (5)	1 (8)

*Note*: Categorical variables are displayed as count (percentage), and continuous variables are displayed as median (range).

Abbreviation: *n*, number.

^a^ In 3 Exp^+^ individuals, European ancestry is self‐reported; the remaining ancestry data were determined by principal component analysis using FRAPOSA (Zhang et al., 2020).

### 3.2 Histopathological categorisation of guttae appearance did not segregate with CTG18.1 expansion status

From the 60 Exp^+^ cases, 53 were assigned ‘typical’, two ‘intermediate’, and five ‘atypical’ by an ocular histopathologist (Figure S2, Table 2). From the 12 Exp^−^ cases, 10 were assigned ‘typical’, one ‘intermediate’, and one ‘atypical’. All but 2/60 (3%) of Exp^+^ samples showed guttae (Figure S3). Epidemiological characteristics are presented in Table 3.

**TABLE 2:** Genotype–phenotype correspondence.

Guttae category	Typical	Not typical		Total
		Intermediate	Atypical	
Genotype				
Exp^+^ FECD	53	2	5	60
Exp^−^ FECD	10	1	1	12
Total	63	3	6	72

*Note*: The guttae category was assigned by an experienced ocular histopathologist according to the definition in Figure 1.

Abbreviations: Exp^+^, expansion‐positive; Exp^−^, expansion‐negative; FECD, Fuchs endothelial corneal dystrophy.

**TABLE 3:** Participant characteristics by guttae category: ‘typical’ versus ‘not typical’ categories.

	Typical	Not typical (intermediate and atypical)	OR (95% CI)	*p* *
Participants, *n* (eyes)	63 (63)	9 (9)		
Female, *n* (%)	46 (73)	2 (22)	0.11 (0.01–0.65)	0.005017
Exp^+^, *n* (%)	53 (84)	7 (78)		n.s. (0.6393)
Ancestry^a^				
European, *n* (%)	51 (81)	8 (89)		n.s.^b^ (1)
African, *n* (%)	2 (3)	1 (11)		
South Asian, *n* (%)	1 (2)	0 (0)		
Not known, *n* (%)	9 (14)	0 (0)		
Age at first keratoplasty, years, median (range)	67 (32–87)	67 (34–86)		
Age at tissue sample collection, years, median (range)	69 (32–87)	67 (34–86)		n.s. (0.5176)
Type of surgery				
Penetrating keratoplasty, *n* (%)	27 (43)	2 (22)		n.s. (0.2975)
Endothelial keratoplasty, *n* (%)	36 (57)	7 (78)		
Keratoplasty only				n.s.^c^ (1)
Phakic throughout surgery, *n* (%)	10 (16)	2 (22)		
Pseudophakic throughout surgery, *n* (%)	22 (35)	2 (22)		
Combined cataract surgery, *n* (%)	30 (47)	4 (44)		
Lens status not documented, *n* (%)	1 (2)	1 (11)		
Year of surgery, median (range)	2014 (1998–2023)	2017 (2010–2019)		n.s. (0.3607)
Additional eye conditions with effect on the cornea before surgery				
None, *n* (%)	54 (86)	3 (33)	0.09 (0.01–0.50)	0.001846
≥1 additional eye condition, *n* (%)	9 (14)	6 (67)		
Keratoconus, *n*	3 (5)	4 (44)	0.07 (0.01–0.51)	0.004208
Glaucoma, *n* (%)	4 (6)	1 (11)		n.s. (0.4761)
Keratitis, *n* (%)	2 (3)	2 (22)		n.s. (0.07361)

Abbreviations: Exp^+^, expansion‐positive; Exp^−^, expansion‐negative; FECD, Fuchs endothelial corneal dystrophy; *n*, number; n.s., not significant.

^a^ In three individuals, European ancestry is self‐reported; the remaining ancestry data was determined by principal component analysis using FRAPOSA (Zhang et al., 2020).

^b^ Values were calculated comparing European and non‐European.

^c^ Values were calculated comparing keratoplasty only versus keratoplasty with combined cataract surgery.

* *p*‐value was calculated either by Fisher's exact test for categorical variables, or Mann–Whitney *U* test for continuous variables after evaluating the data distribution for normality.

When we compared the grading, both raters agreed in 78% of cases (Table 4). Comparing ‘typical’ categorisation, Cohen's kappa was 0.31, indicating a fair agreement. No significant difference was observed between the histopathological categorisation (‘typical’ vs. ‘not typical’) between Exp^+^ and Exp^−^ groups, regardless of the inconsistencies between the rater outcomes (Fisher's exact test, rater 1 [ocular pathologist]: Odds Ratio [OR], 1.5; 95% CI: 0.14–9.7; *p* = 0.6393; rater 2 [ophthalmologist]: OR, 0.9; 95% CI: 0.14–4.32; *p* = 1). There was only a small or negligible effect size in the histopathological categorisation between Exp^+^ and Exp^−^ cases for rater 1 and 2 respectively (Cohen's *h*, rater 1: 0.14; 95% CI: −0.17 to 0.45; rater 2: −0.04; 95% CI: −0.35 to 0.27). We repeated this analysis excluding all cases with a comorbidity of KC (*n* = 7). No significant difference was observed in the histopathological categorisation (‘typical’ vs. ‘not typical’) between Exp^+^ and Exp^−^ groups for both raters.

**TABLE 4:** Inter‐rater reliability.

		Guttae category	Rater 1 (experienced ocular histopathologist)			Total
			Typical	Not typical		
				Intermediate	Atypical	
Rater 2 (ophthalmologist)		Typical	50	2	1	53
	Not typical	Intermediate	8	1	0	9
		Atypical	5	0	5	10
		Total	63	3	6	72

*Note*: The guttae category was assigned according to the definition in Figure 1.

### 3.3 Guttae appearance was consistent between contralateral FECD corneal tissue pairs

**FIGURE 2:**
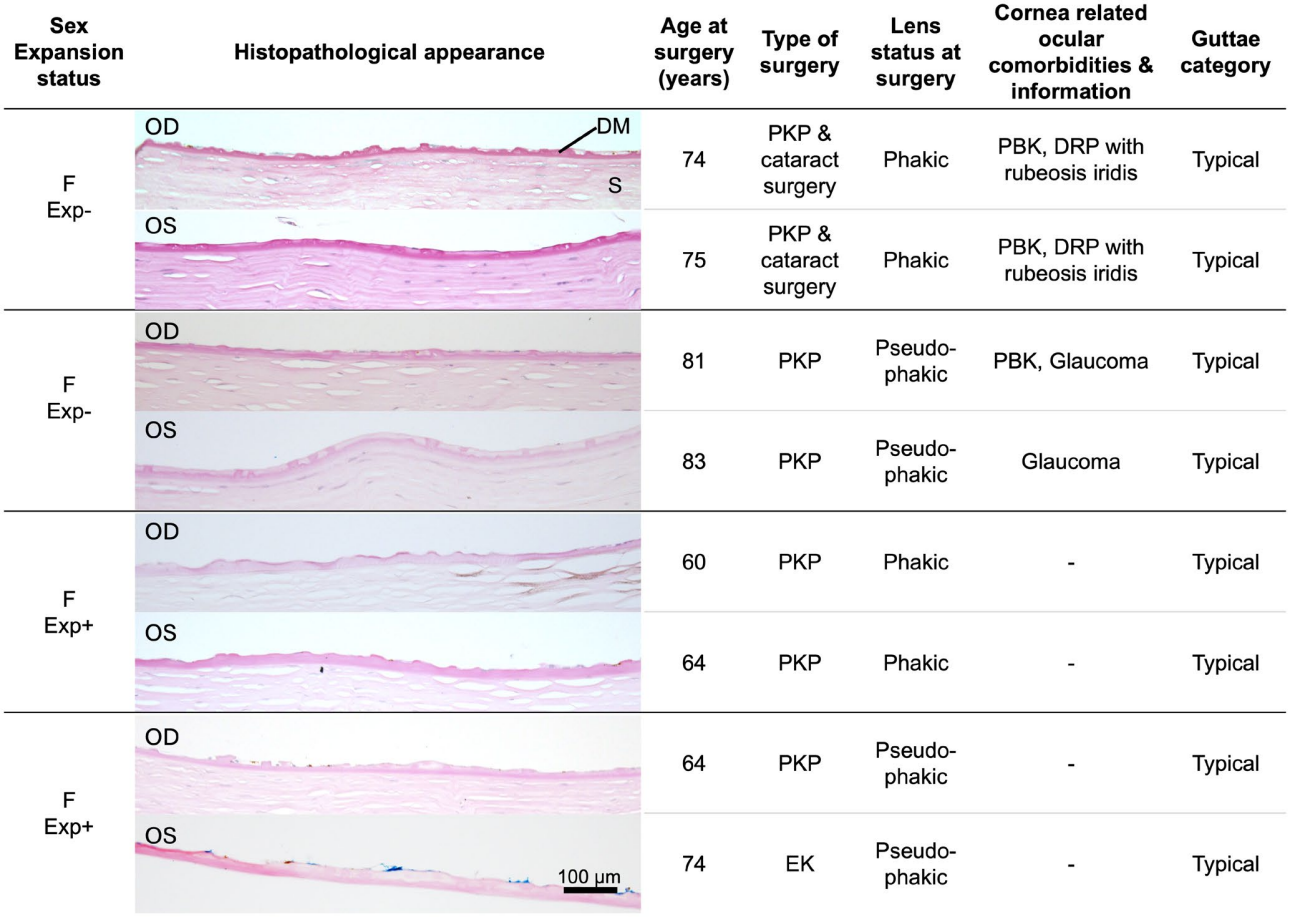
Contralateral eye comparison revealed no differences in categorisation and overall histological appearance of guttae. Contralateral corneal tissue was available from four unrelated FECD cases. Periodic acid‐Schiff‐stained sections of corneal stroma (S), Descemet membrane (DM), and endothelium. Periodic acid‐Schiff, 20× objective magnification. Scale bar = 100 μm. DRP, diabetic retinopathy; EK, endothelial keratoplasty; Exp^−^, expansion‐negative; Exp^+^, expansion‐positive; F, female; FECD, Fuchs endothelial corneal dystrophy; OD, right eye; OS, left eye; PBK, pseudophakic bullous keratopathy; PKP, penetrating keratoplasty.

Bilateral corneal tissue samples were available from four patients (two Exp^+^, two Exp^−^). The interval between these corneal surgeries ranged from 1 to 10 years. Histopathological categorisation of guttae and the overall histological appearances were concordant in all pairs (Figure 2).

### 3.4 Histopathological categorisation appeared to segregate with sex and co‐occurrence of keratoconus

**FIGURE 3:**
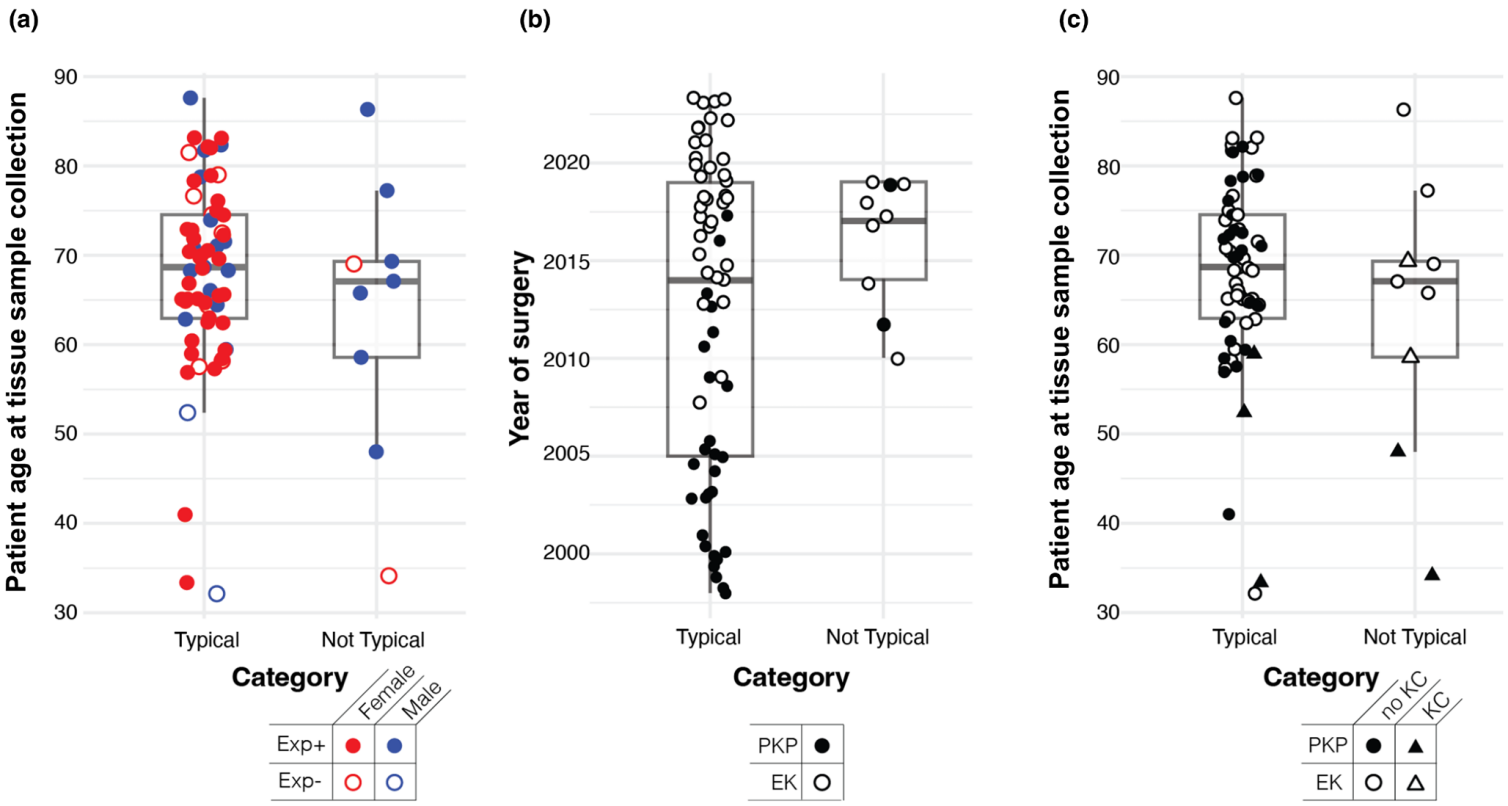
Epidemiological characteristics by histopathological categorisation. (a) CTG18.1 expansion‐positive samples were observed in both the ‘typical’ and the ‘not typical’ categories. However, there was a higher proportion of males in the ‘not typical’ category (uncorrected exploratory *p*‐value, *p* = 0.005017). (b) In the ‘not typical’ category, all surgeries were performed from 2010 onwards, in contrast to cases in the ‘typical’ category, which included surgeries spanning more than two decades. (c) There was no difference in age between individuals in both categories (uncorrected exploratory *p*‐value, *p* = 0.518). The proportion of FECD individuals with a comorbidity of KC was relatively higher in the ‘not typical’ group than in the ‘typical’ group (uncorrected exploratory *p*‐value, *p* = 0.004208). EK, endothelial keratoplasty; Exp^+^, expansion‐positive; Exp^−^, expansion‐negative; FECD, Fuchs endothelial corneal dystrophy; KC, keratoconus; PKP, penetrating keratoplasty.

Specimens in the ‘not typical’ group were more likely to be from males (7/9 vs. 17/63, OR, 0.11; 95% CI: 0.01–0.65; uncorrected exploratory *p*‐value, *p* = 0.005017) and were significantly more often affected by KC (4/9 vs. 3/63; OR, 0.07; 95% CI: 0.01–0.54; uncorrected exploratory *p*‐value, *p* = 0.004208) (Figure 3a,c, Table 3). We repeated the analysis for histopathological categorisation segregating by sex after excluding all cases with a comorbidity of KC and continued to observe the enrichment of males in the ‘not typical’ category (9/10 vs. 11/55 males, OR, 0.09; 95% CI: 0.00–1.05; uncorrected exploratory *p*‐value, *p* = 0.02827). However, we did not adjust for multiple tests for all the above analyses, and results should be interpreted cautiously.

Patient's age at tissue sample collection did not differ significantly between categorical groups (‘typical’: 69 years [32–87], ‘not typical’: 67 years [34–86], uncorrected exploratory *p*‐value, *p* = 0.518 [Mann–Whitney *U* test]) (Figure 3c). The proportion of PKP and EK also did not differ significantly between groups. Two of the nine ‘not typical’ cases originated from PKP, while seven were from EK (Figure 3b, Table 3). All ‘not typical’ cases came from 2010 onwards when EK replaced PKP as the treatment of choice for FECD in the UK (Figure 3b). Among the ‘typical’ cases, however, the proportion of PKP (27/63) and EK (36/63) was more balanced. The proportion of the other non‐significantly different characteristics is listed in Table 3.

## 4 DISCUSSION

This study represents the largest genetically refined histopathological study of FECD to date, to the best of our knowledge. Overall, we found no correlation between CTG18.1 expansion status and the appearance and density of guttae on the DM of individuals with FECD. We also explored whether additional histological and clinical parameters segregated with the differences in guttae and found that unusual guttae appearance might potentially be influenced by sex and coexisting keratoconus.

Our recent transcriptomic study (Bhattacharyya et al., 2024) highlighted that CTG18.1 expansions exert a distinctive transcriptomic signature of dysregulation in CECs, including the dysregulation of numerous extracellular matrix‐associated genes. Given these findings and the fact that guttae are the most prominent histological characteristic of FECD (Matthaei et al., 2019), we decided to focus on guttae appearance in this study. However, we did not observe overt structural changes in guttae observable by light microscopy and standard histopathological staining. Although the defined phenotypic categories did not seem to correspond with the genotype, we cannot rule out an effect given the limited sample size of the cohort. Other histopathological hallmarks also contribute to the FECD phenotype (Matthaei et al., 2019; Petrela & Patel, 2023), which may differ depending on the genetic underlying cause of FECD (Bhattacharyya et al., 2024), as is seen in cases with causative *COL8A2* pathogenic variants (Gottsch, Sundin, et al., 2005; Gottsch, Zhang, et al., 2005). The lack of relationship between guttae appearance and CTG18.1 expansions in *TCF4* observed in this study might be explained by the fact that *TCF4* does not encode for a structural component of DM, unlike *COL8A2*. Further investigations employing advanced microscopy methods to further explore the guttae and/or to observe structural differences across the DM (Brockmann et al., 2014; Weller et al., 2024) may shed light on the CTG18.1 expansion genotype–phenotype relationship.

The higher prevalence of male participants with a ‘not typical’ categorisation might be linked to the known role of oestrogen in the pathway underlying FECD (Kumar et al., 2024; Liu et al., 2020). The high proportion of cases with other eye conditions, especially KC, suggests that other corneal diseases may influence the appearance of guttae. It is also likely that, in cases with concurrent KC, the primary indication for surgery was the KC, while the FECD, though clinically present, was less advanced. In earlier studies, no differences were reported in combined cases of FECD and KC (Cremona et al., 2009; Liu et al., 2023). However, our findings suggest that further investigation of the relationship between KC, sex, and guttae morphology is warranted.

Another major contributor to the guttae phenotype is the stage of the disease. Numerous studies have stated that guttae density and dimension correlate with disease severity (Kocaba et al., 2018; Ong Tone et al., 2021; Son et al., 2014) with only one conflicting study (Bourne et al., 1982). However, despite the interval of up to 10 years, the contralateral similarity in guttae appearance observed in our study suggests that disease progression may not be a major factor, assuming a similar disease progression in both eyes. Furthermore, the comparable age of patients undergoing keratoplasty with ‘typical’ and ‘not typical’ guttae appearance indicates that the difference cannot be attributed solely to disease progression. Studies have also shown that guttae seen clinically in retroillumination are not necessarily associated with histopathological guttae (Hogan et al., 1974; Gottsch, Sundin, et al., 2005; Gottsch, Zhang, et al., 2005; Frauches et al., 2024). Indeed, we saw two Exp^+^ specimens with no guttae. Though misdiagnosis of these individuals cannot be ruled out, a possible explanation is that clinical guttae can result from the distortion of light going through the irregularly shaped collagen bundles of the DM and not by the guttae themselves (Magovern et al., 1979; Gottsch, Sundin, et al., 2005; Gottsch, Zhang, et al., 2005), or due to pseudoguttae from oedema (Doughty et al., 2011; Krachmer et al., 1981), which would not be observed histopathologically. The histopathological differences may also be influenced by exogenous or environmental factors, like oxidative stress and ultraviolet light (Jurkunas et al., 2010; Ong Tone et al., 2021), as well as surgery‐related factors. As DM alterations can also be linked to age (Matthaei et al., 2019), it is important to note that there was no significant difference in age between the two groups compared in this study.

In conclusion, this study is the first attempt to correlate CTG18.1 expansion status with the histopathology of FECD. Although there was no correlation with expansion status, we have developed a scoring system to classify histopathological guttae appearance, which can be applied in future work. Based on these findings, further investigations considering other clinical measurement techniques, detailed medical history for exogenous factors, the inclusion of early‐stage patients, and a larger overall cohort size will be needed to clarify what is driving phenotypic differences seen in guttae. Continuing research on phenotypic variations associated with different genotypes could provide new insights into FECD clinical hallmarks and aid the advancement of gene‐directed therapeutic development (Angelbello et al., 2021; Hu et al., 2018; Powers et al., 2022; Zarouchlioti et al., 2018).

## FUNDING INFORMATION

Supported by a UKRI Future Leader Fellowship MR/S031820/1; and MR/Y019911/1 (AED), Moorfields Eye Charity GR001337 (AED), Sight Research UK SEE016 (NB), the National Institute for Health Research Biomedical Research Centre at Moorfields Eye Hospital National Health Service Foundation Trust, UCL Institute of Ophthalmology NIHR203322, the DFG (research fellowship by the German Research Foundation – project number 527928847 [ASK]) and Fight for Sight 5171/5172 (AED). The funding sources had no role in the design or conduct of this research. The authors alone are responsible for the content and writing of the paper.

## CONFLICT OF INTEREST STATEMENT

AS Kladny, N Bhattacharyya, M Abreu Costa, SJ Tuft, C Thaung, none; AE Davidson has previously acted as a paid consultant for Triplet Therapeutics Ltd, LoQus23 Therapeutics Ltd, Design Therapeutics Ltd, and had a research collaboration with ProQR Therapeutics. AE Davidson has an ongoing research collaboration with Prime Medicine.

## Supporting information


Figure S1.

Figure S2.

Figure S3.
