# Supplementary material for: Exploring the histopathological signature of repeat‐mediated Fuchs endothelial corneal dystrophy
Source: Acta Ophthalmol. 2025 Oct 14;104(3):333–41. doi: 10.1111/aos.70014 (PMC13058678; doi:10.1111/aos.70014)
Supplement: Supplementary file 1 — Figure S1. [file AOS-104-333-s001.pdf]

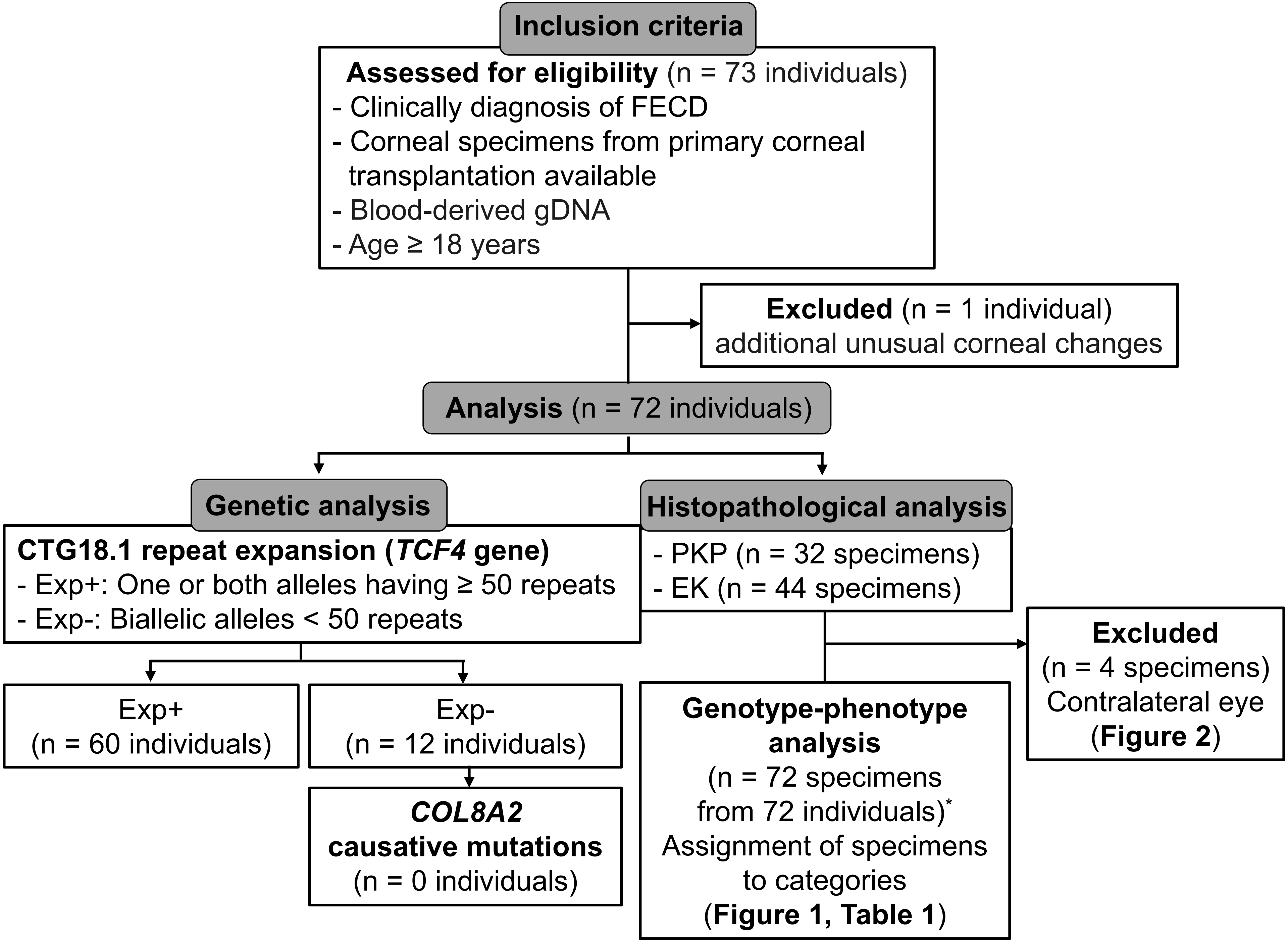
Inclusion criteria
Assessed for eligibility (n = 73 individuals)
- Clinically diagnosis of FECD
- Corneal specimens from primary corneal transplantation available
- Blood-derived gDNA
- Age ≥ 18 years
Excluded (n = 1 individual) additional unusual corneal changes
Analysis (n = 72 individuals)
Genetic analysis
CTG18.1 repeat expansion (TCF4 gene)
- Exp+: One or both alleles having ≥ 50 repeats
- Exp-: Biallelic alleles < 50 repeats
Exp+ (n = 60 individuals)
Exp- (n = 12 individuals)
COL8A2 causative mutations (n = 0 individuals)
Histopathological analysis
- PKP (n = 32 specimens)
- EK (n = 44 specimens)
Excluded (n = 4 specimens) Contralateral eye (Figure 2)
Genotype-phenotype analysis (n = 72 specimens from 72 individuals)*
Assignment of specimens to categories (Figure 1, Table 1)
