# Supplementary figures and images for: Exploring the histopathological signature of repeat‐mediated Fuchs endothelial corneal dystrophy
Source: Acta Ophthalmol. 2025 Oct 14;104(3):333–41. doi: 10.1111/aos.70014 (PMC13058678; doi:10.1111/aos.70014)

| Category |     | Genotype |                                                                                      | Typical                                                                               | Atypical |
|----------|-----|----------|--------------------------------------------------------------------------------------|---------------------------------------------------------------------------------------|----------|
| Genotype |     |          |                                                                                      |                                                                                       |          |
| Exp+     | EK  |          | 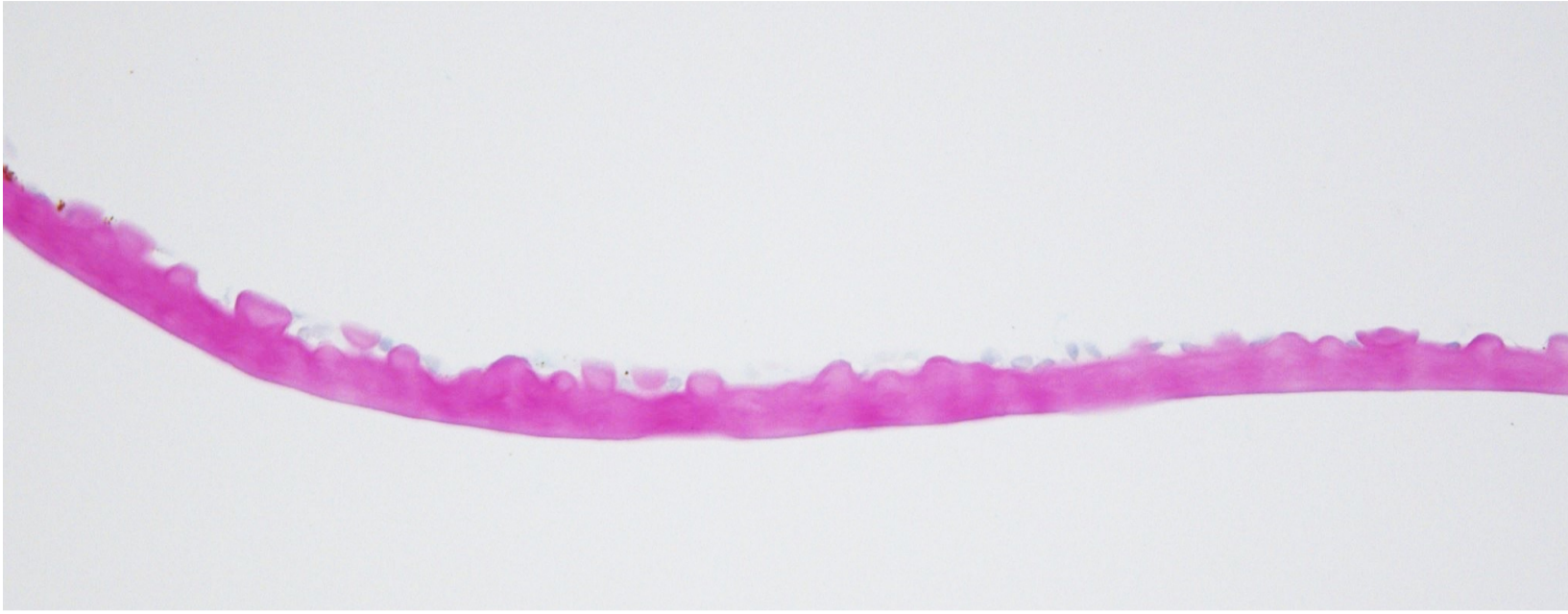   | 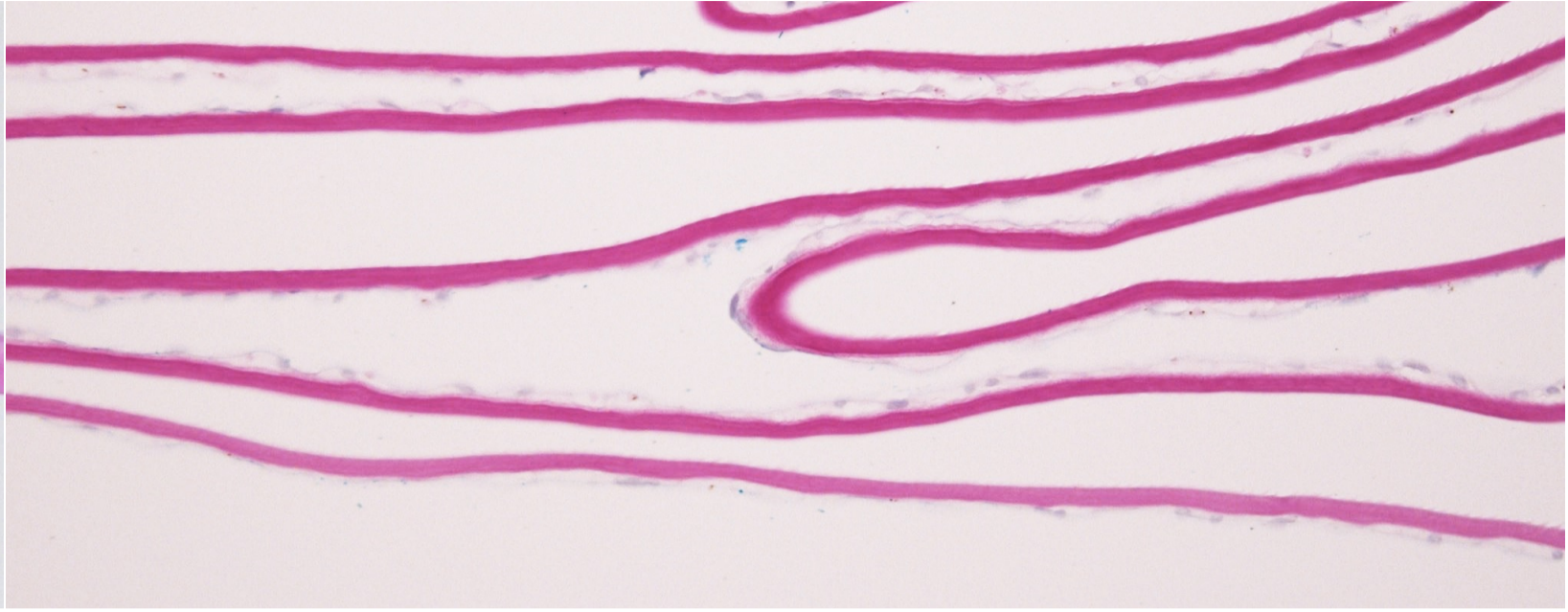   |          |
|          | PKP |          | 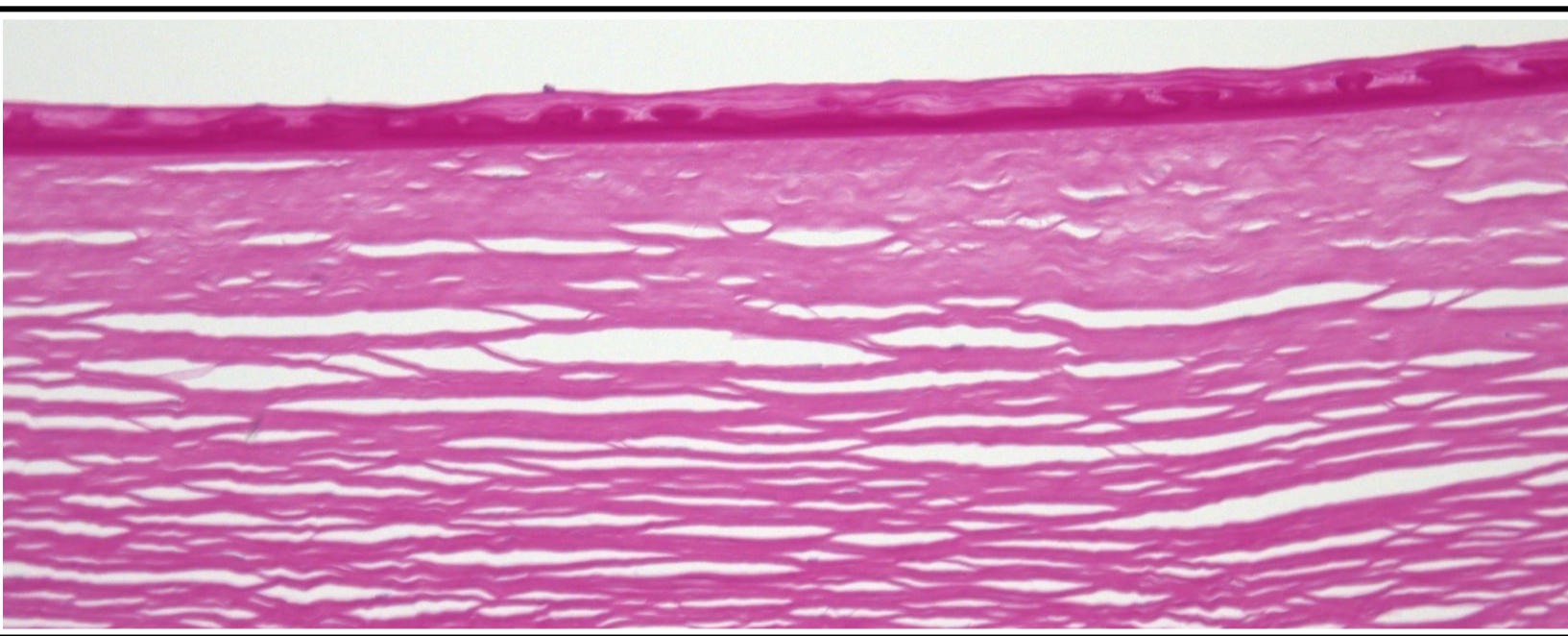  | 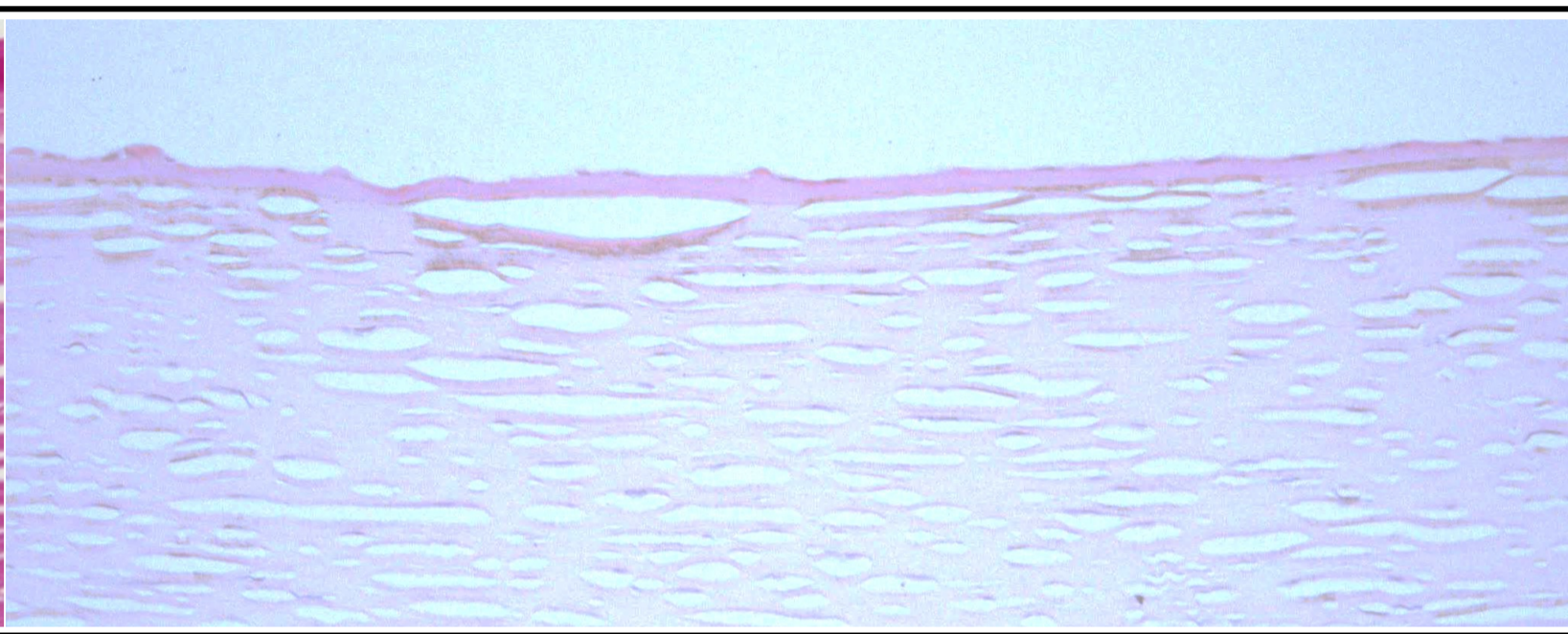  |          |
| Exp-     | EK  |          | 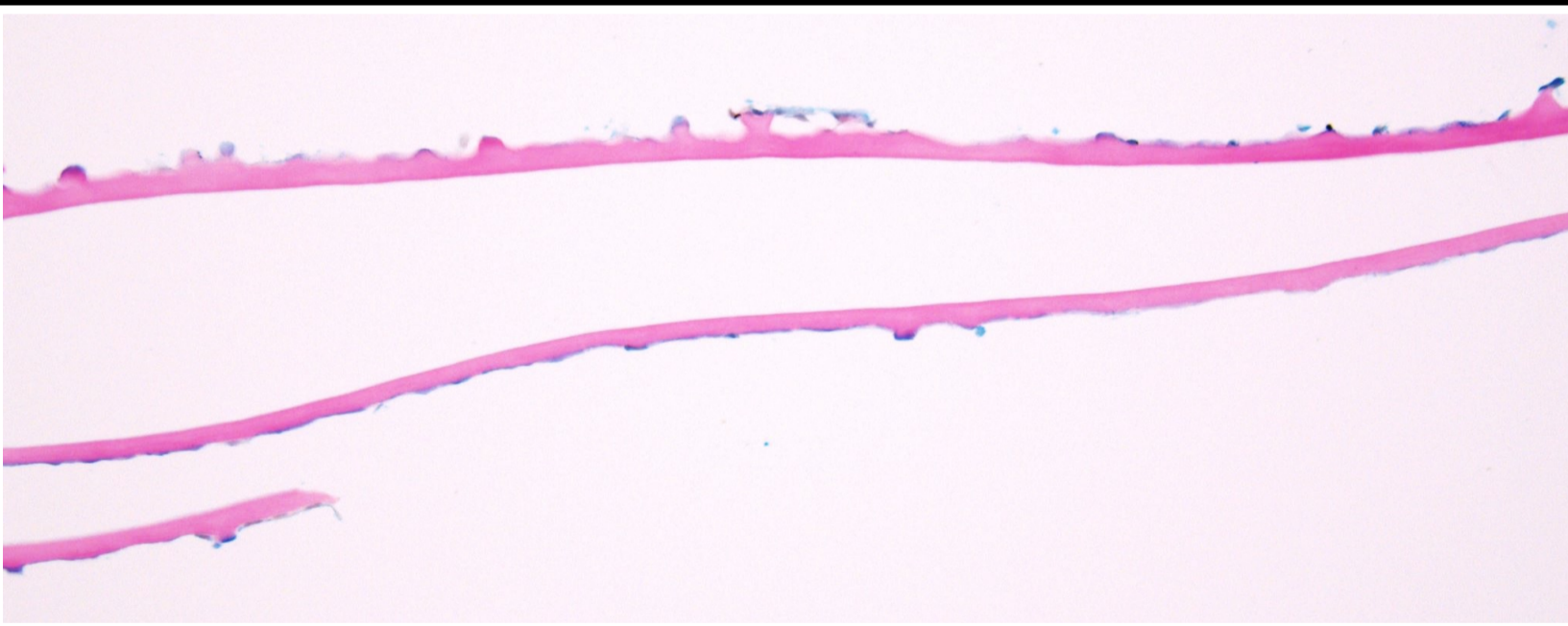 | 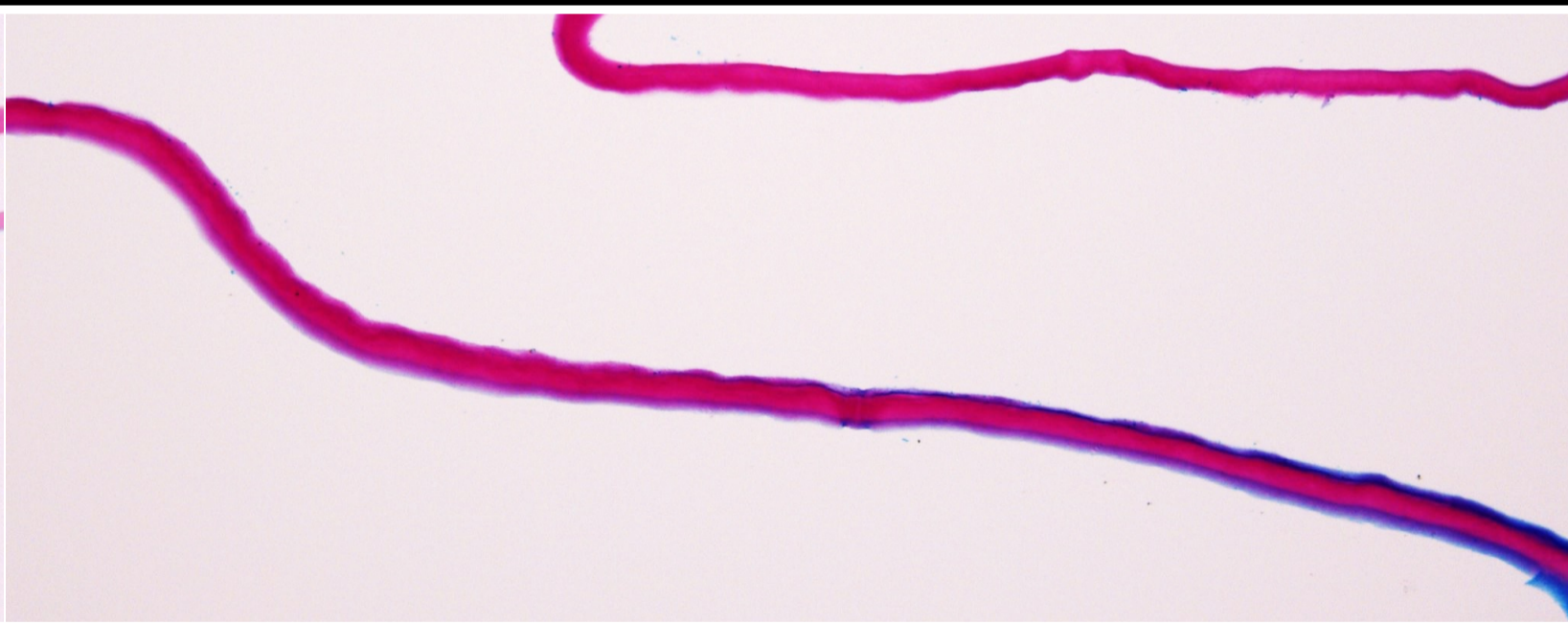 |          |
|          | PKP |          | 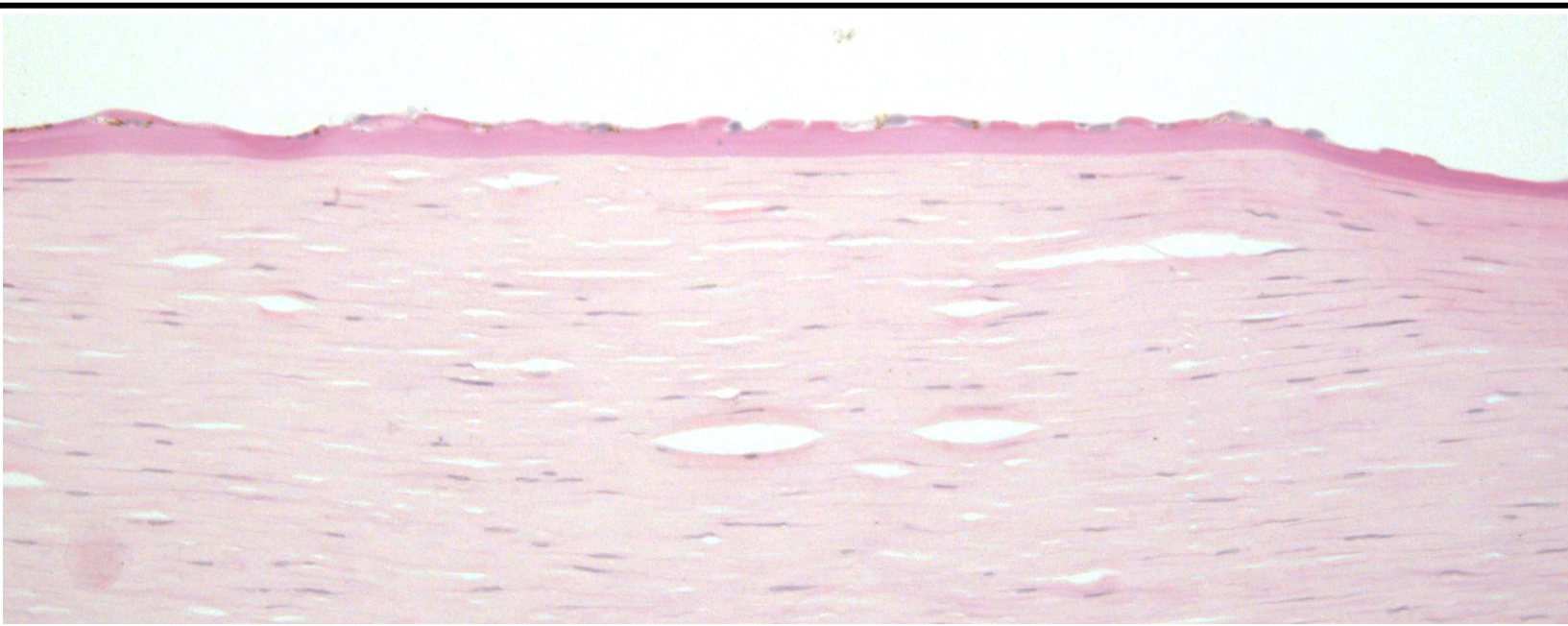 | 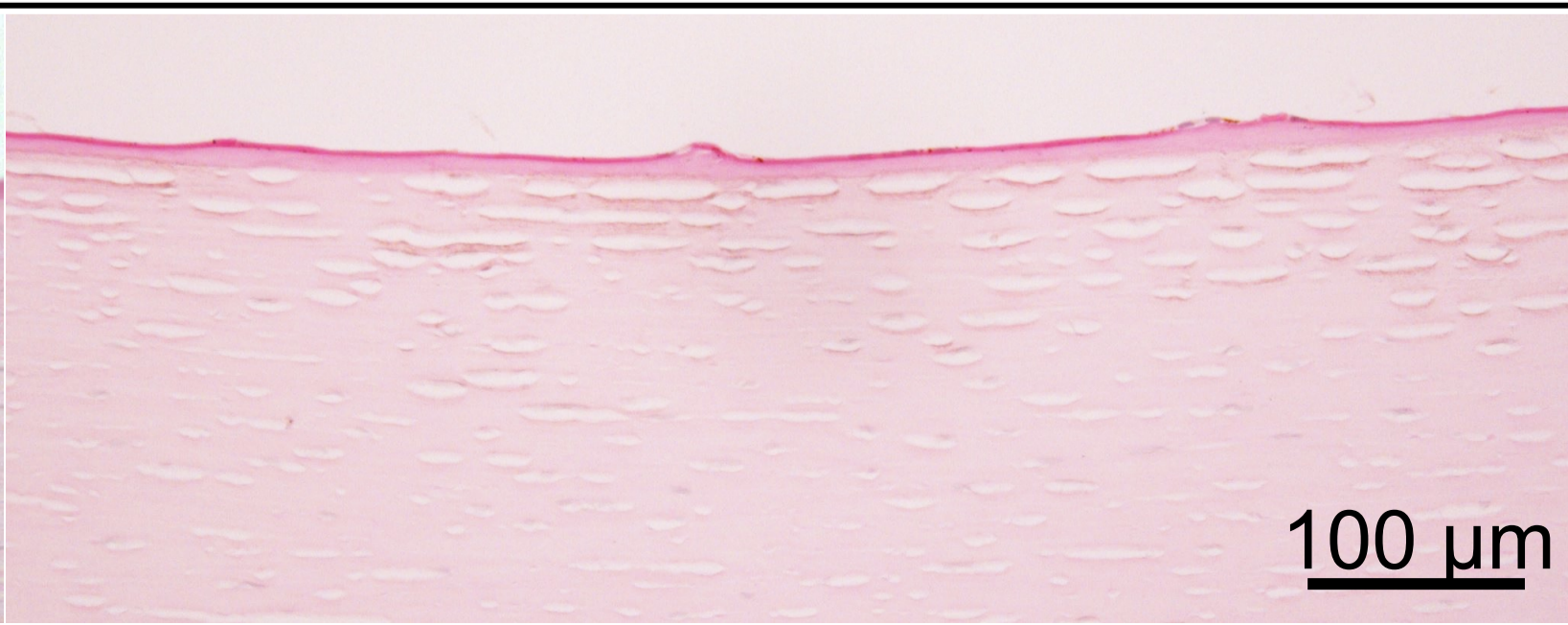 |          |

100 μm

100  $\mu$ m

Supplement: Supplementary file 2 — Figure S2. [file AOS-104-333-s002.pdf]
