# Supplementary material for: Exploring the histopathological signature of repeat‐mediated Fuchs endothelial corneal dystrophy
Source: Acta Ophthalmol. 2025 Oct 14;104(3):333–41. doi: 10.1111/aos.70014 (PMC13058678; doi:10.1111/aos.70014)
Supplement: Supplementary file 3 — Figure S3. [file AOS-104-333-s003.pdf]

Sex Expansion status	Histopathological appearance	Age at tissue sample collection (years)	Type of surgery	Lens status at tissue sample collection	Ocular comorbidities & information	Guttae category
M Exp+	OD 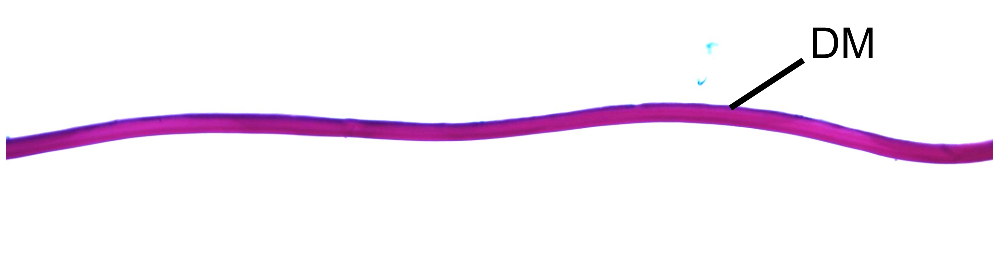 Stained with PAS	66	EK & cataract surgery	Phakic	Bullous keratopathy with cystic oedema especially in the inferior third of both corneas, serology negative for active herpes endotheliitis	Atypical
M Exp+	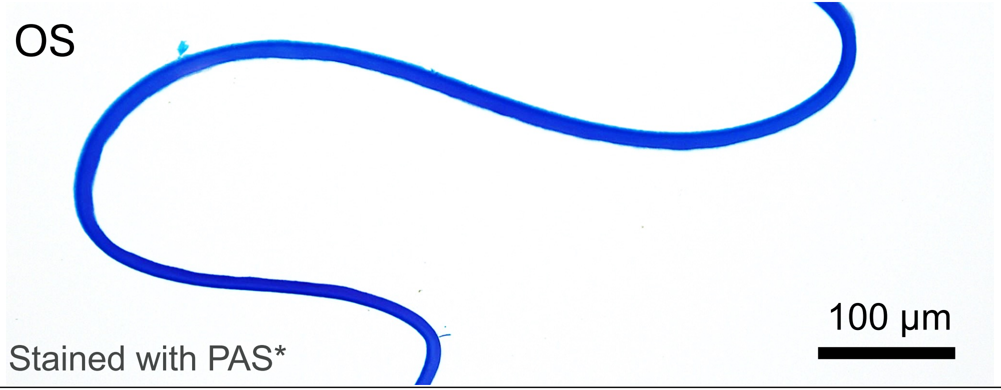 Stained with PAS*	77	EK & cataract surgery	Phakic	Enucleation of partner eye after choroidal melanoma	Atypical
